# 64. Novel Biomarkers Improve Estimation of Vancomycin Clearance in Critically Ill Children

**DOI:** 10.1093/ofid/ofab466.064

**Published:** 2021-12-04

**Authors:** Kevin J Downes, Athena F Zuppa, Anna Sharova, Lauren Gianchetti, Emily Duffey, Stuart L Goldstein, Michael Neely

**Affiliations:** 1 Children’s Hospital of Philadelphia, Philadelphia, PA; 2 Cincinnati Children’s Hospital Medical Center, Cincinnati, Ohio; 3 Children Hospital Los Angeles, Los Angeles, California

## Abstract

**Background:**

There are a paucity of robust population PK (popPK) models to inform vancomycin (VAN) dosing in critically ill children. The majority of published models incorporate peak/trough data and rely on flawed estimates of renal function. We sought to develop a popPK model for IV VAN in critically ill children utilizing novel plasma and urinary biomarkers.

**Methods:**

We conducted a prospective observational study of critically ill children prescribed VAN for a suspected infection in the CHOP pediatric ICU. Children < 1 year of age and those receiving ECMO or CRRT were excluded. Five VAN samples were collected from a single dosing interval for each subject. Plasma biomarkers (creatinine [Cr], cystatin C [CysC], NGAL) and urinary biomarkers (CysC, NGAL, KIM-1, osteopontin) were collected the morning of PK sampling; urinary biomarkers were corrected for urine creatinine. Nonparametric popPK modeling was performed using Pmetrics. The impact of renal function (GFR) on VAN clearance (CL) was estimated first, comparing model performance with each biomarker (Cr and plasma CysC). The influence of age, sex, additional biomarkers, PIM3 score, and receipt of vasopressors as covariates was then assessed for relevant PK parameters.

**Results:**

30 subjects completed the study. Median age was 10 years (range 1-17); 76% were male. The majority (90%) of children received VAN for suspected sepsis. PK sampling occurred at a median of 37.7 hours (range 24.6-94.8) into VAN treatment; 136 VAN samples were included. A 2-compartment model with fixed allometric scaling of 0.75 on clearances and 1 on volumes best described the data. CysC-based GFR as a covariate on VAN CL using the HOEK formula (GFR = -4.32 + (80.35/CysC)) resulted in the best model fit. Age and plasma NGAL were also informative on VAN CL in the final model (**Figure 1**). During model building, urinary NGAL was also associated with VAN CL (comparable to plasma NGAL) and outperformed Cr, although it was not retained in the final model.

Figure 1. Final population PK model and parameter estimates.

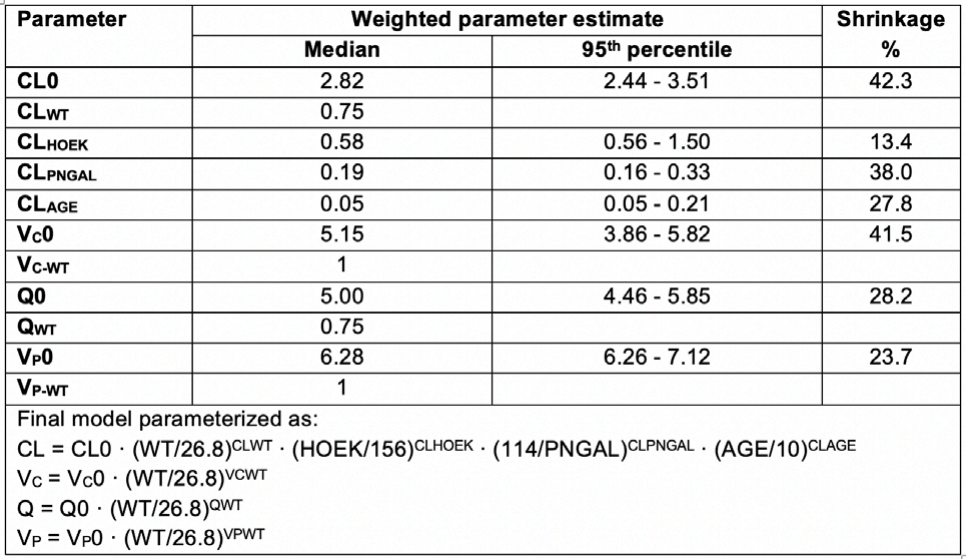

**Conclusion:**

Plasma CysC is a better renal function estimate than Cr to inform VAN clearance in critically ill children. Urinary and plasma NGAL also improved estimation of VAN CL during popPK modeling. Novel biomarkers can better describe VAN exposures in critically ill children than reliance on Cr alone.

**Disclosures:**

**Kevin J. Downes, MD**, Merck (Individual(s) Involved: Self): Grant/Research Support **Stuart L. Goldstein, MD**, **Bioporto** (Consultant, Grant/Research Support)

